# Implementation of major trauma app: usability and data completeness

**DOI:** 10.1186/s12873-024-01022-w

**Published:** 2024-07-29

**Authors:** Miss Joanna Butler, Clare Arneil, Alan S. Whitelaw, Kevin Thomson, Malcolm W. G. Gordon, Josh Thorburn, Darren Shiels, David J. Lowe

**Affiliations:** 1 Emergency Department, Queen Elizabeth University Hospital, Glasgow, G52 4TF UK; 2 School of Health and Wellbeing, University of Glasgow, Glasgow, G12 8RZ UK; 3 Clyde Trauma and Orthopaedics, Royal Alexandra Hospital, Paisley, PA2 9PN UK

**Keywords:** Trauma, Electronic clinical decision making, Documentation, Usability

## Abstract

**Background:**

The current UK standard for major trauma patients is to record notes in a paper trauma booklet. Through an innovative collaboration between a major trauma centre and a digital transformation industry partner, a TraumaApp was developed. Electronic notes have been shown to have fewer errors, granular data collection and enable time stamped contemporaneous record keeping. Implementation of digital clinical records presents a challenge within the context of trauma multidisciplinary trauma resuscitation. Data can be easily accessible and shared for quality improvement, audit and research purposes. This study compared paper and electronic notes for completeness and for acceptability data following the implementation of the TraumaApp.

**Methods:**

Trauma team members who performed scribe function attended training for the newly launched TraumaApp. Two staff members acted as scribe, using either the paper trauma booklet or TraumaApp, and attended major trauma calls. A framework for comparison of paper and electronic notes was created and used for a retrospective review of major trauma patients’ notes. Statistical analysis was performed using a two-tailed t-test. Staff using the TraumaApp completed a System Usability Score questionnaire.

**Results:**

There was a total of 37 data points for collection per case. The mean numbers collected were paper notes 24.1 of 37 (65.1%) and electronic notes, 25.7 of 37 (69.5%). There was no statistical significance between the completeness of paper and electronic notes. The mean System Usability Score was 68.4.

**Discussion:**

Recording accurate patient information during a major trauma call can be challenging and the role of the scribe to accurately record events is critical for immediate and future care. There was no statistically significant difference in completeness of paper and electronic notes, however the mean System Usability Score was 68.4, which is greater than the internationally validated standard of acceptable usability.

**Conclusion:**

It is feasible to introduce digital data collection tools enabling accurate record keeping during trauma resuscitation and improve information sharing between clinicians.

**Supplementary Information:**

The online version contains supplementary material available at 10.1186/s12873-024-01022-w.

## Background

Trauma remains the fourth leading cause of death in Western countries, with approximately 1000 cases per year in Scotland described as major trauma [1]. The West of Scotland has a population of 2.7 million people and the Scottish Trauma Network (STN) encompasses four areas, each with a central Major Trauma Centre (MTC). The Queen Elizabeth University Hospital (QEUH) in Glasgow was designated as MTC for the West of Scotland in August 2021 [2].

Restructuring of trauma provision into networks in the UK led to centralisation of resource, with the goal of improving outcomes in patients, but requiring significant investment. To demonstrate impact, national audit groups such as the Trauma Audit and Research Network (TARN) England, and the Scottish Trauma Audit Group (STAG) in Scotland, require high quality data to support insights to improve delivery, future policy and investment [3].

In the post-pandemic era of the NHS, there has been an accelerated transition into digitally aided care provision [4]. There is an aim to develop electronic data collection systems which could facilitate the collection of data [5].

Paper notes are readily available and can be edited contemporaneously, but they can also be inaccurate and challenging to navigate [6]. Structured clinical data collected on digital tools can be more readily organised and easily accessed but requires development of a context specific user interface to reflect often complex clinical pathways of care. Comparison studies of paper and electronic notes have shown that electronic notes have fewer omission errors, are equally as accurate as paper notes, can capture up to 24% more data, and improve communication between specialist teams [6, 7].

Current International and UK standard is to record trauma patient notes in a paper trauma booklet. Trauma resuscitation is fast paced, with team members often performing concurrent tasks and parallel activities; monitoring all of these can be challenging [8]. Often there is a large volume of data points generated within a short space of time and this can be difficult to document accurately and completely [9]. Particularly, some clinical interventions require clear documentation of performance time, such as application and removal of tourniquets. This has been shown to be inconsistently documented in paper notes [8]. Completion of trauma booklets is allocated to a scribe who may be a member of nursing, medical staff or, in some centres, trauma coordinators. At the end of each case, paper trauma booklets must be collated with any separate documentation, such as prescription charts or specialty notes, and either kept with the patient or scanned. This risks loss of records which, accompanied by illegible handwriting and transcription errors, leads to inaccurate documentation [9].

In Emergency Departments (EDs), there has been reluctance to use electronic documentation due to high patient turnover and concerns about the increased burden on staff who are unfamiliar with these systems [10]. Studies have commented that frequent tab switching and problems navigating user interfaces can result in delayed data capture [11]. This is supported by military studies conducted in combat trauma centres which state that completion of electronic documentation, compared with paper, was statistically significantly higher [9].

In response to these challenges, a TraumaApp project was initiated to create a digital data collection tool to record the ED care for major trauma patients [12]. The aim for this app was to improve speed, accuracy and completeness of documentation during initial major trauma resuscitation and management. The multi-disciplinary development team consisted of members of the QEUH research team, clinicians representing the Scottish Trauma Network and Daysix, a digital transformation company. The project was funded by InnovateUK and hosted by the West of Scotland Innovation Hub as part of their triple helix approach combining the NHS, academia and industry partners. Using National Institute for Health and Care Excellence consensus guidelines for complete documentation, a novel TraumaApp was developed [13, 14].

## Aim

The aim of this preliminary study was to compare the use of digital data collection tool (TraumaApp) against the current paper trauma booklet used in the ED at the QEUH, focussing on data completeness and usability.

## Methods

This mixed methodology study ran from August to October 2021 in the Queen Elizabeth University Hospital, Glasgow. The centre sees approximately 110,000 attendances per year and 850 major trauma activations for patients aged > 16 years. The study team comprised three Clinical Fellows in Trauma (CA, DS, JT) who provided training sessions on the Trauma App for staff, using a blended approach of online content, face to face teaching and simulation sessions.

The study was performed during the implementation phase of the TraumaApp (DaySix, Edinburgh, UK) [12], during live major trauma cases. Two scribes were present and would decide between them who would use each of the paper trauma booklet or the TraumaApp; allocation was at the scribes’ discretion. Both scribes recorded information reported by the trauma team. Both paper and electronic notes were uploaded to the electronic health record (EHR) on discharge from the ED.

Cases were identified on retrospective review of EHR using the search parameters of major trauma patients aged > 16 years attending the QEUH ED between 23/08/21 and 03/10/21 inclusively (DT, CA, DS). A framework of key data points was identified for each section of the initial trauma resuscitation: preparations, handover, history and primary survey (see appendix). Both paper and electronic notes were analysed for completeness of each section, creating an error count for missing key data points.

All data was descriptively analysed with a two-tailed t test for comparison of completeness. It was performed on Microsoft Excel.

All staff who used the TraumaApp completed a System Usability Score (SUS) questionnaire, a 10-question tool designed to reliably measure usability [15, 16].

## Results

### Demographics

The demographics of the cases identified on retrospective search are laid out in Table 1. They had a mean age of 48, with a range of 19 to 84 and were 70.5% male. The most frequent mechanism of injury was a fall and Injury Severity Scores (ISS) ranged from 1 to 75. STAG data from 2021 shows a mean age of 61, with a male preponderance of 53.7%. 18.9% of patients had an ISS of greater than 15, showing that our cohort had a generally lower ISS, was older and had a higher proportion of male patients than the group it aims to represent [17].

There was limited documentation of scribe using the TraumaApp, and the majority of paper scribes were Major Trauma Coordinators (9 of 17).

**Table 1:** Trauma case demographics

Case	Team Leader Grade	Mechanism of Injury	Trauma Tier	ISS Score	Scribe Paper	Scribe App
1	Consultant	Fall	2	13	NR	NR
2	Registrar	Fall	NR	9	Reg	SN
3	Registrar	Fall	1	2	MTC	NR
4	Consultant	Fall	1	10	SN	NR
5	Consultant	Fall	NR	3	NR	NR
6	Speciality Doctor	Fall	1	13	MTC	NR
7	Consultant	Assault	1	12	MTC	NR
8	Registrar	RTC	1	13	MTC	NR
9	Consultant	RTC	NR	8	NR	NR
10	Consultant	RTC	2	9	MTC	NR
11	Registrar	Fall	1	20	StudN	NR
12	Consultant	Fall	2	75	NR	NR
13	Consultant	RTC	1	1	MTC	NR
14	Consultant	Fall	1	13	SN	NR
15	Consultant	RTC	1	12	MTC	MTC
16	Consultant	Fall	2	8	MTC	NR
17	Registrar	RTC	1	6	MTC	NR

### Primary analysis

The mean number of data points collected on paper notes was 24.1, and on electronic notes was 25.7, of a potential 37 key data points per case. A further five data points were measured in the ‘Rapid Sequence Induction’ section of the app, but only one case required this intervention. It was therefore excluded from the data analysis. The mean percentage completeness for paper notes was 65.1% (range 13.5–81%) and for electronic notes was 69.5% (range 48.6–86.5%).

**Fig. 1:**
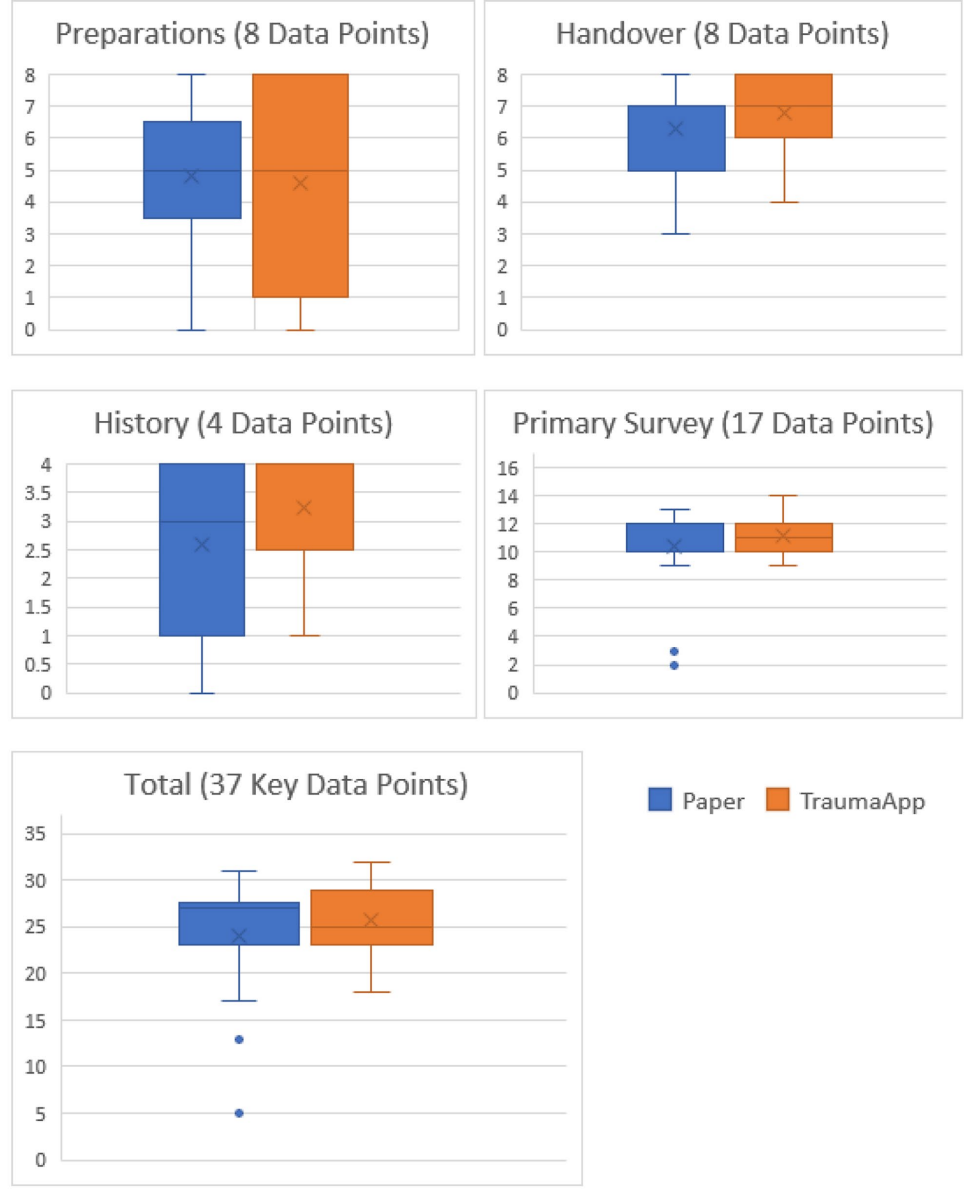
Data completeness – paper versus TraumaApp

A breakdown of the sections and the total completeness of all 37 key data points is shown in Fig. 1.

Analysis of data is shown in Table 2. With a significant p-value of 0.05 there was no statistically significant difference between the completeness of paper versus TraumaApp documentation.

**Table 2:** Statistical analysis - comparison of completeness – Paper versus TraumaApp.

	Mean		Standard Deviation		95% Confidence Interval	*P* value
	Paper	TraumaApp	Paper	TraumaApp		
Preparation	4.82/8	4.59/8	2.32	3.47	-1.90 to 2.37	0.8180
Handover	6.29/8	6.76/8	1.45	1.25	-1.65 to 0.71	0.4102
History	2.59/4	3.24/4	1.50	1.09	-1.59 to 0.30	0.1653
Primary Survey	10.35/17	11.12/17	3.12	1.5	-2.33 to 0.80	0.3170
Total	24.06/37	25.71/37	6.63	3.93	-5.79 to 2.49	0.4116

### System usability scores

**Fig. 2:**
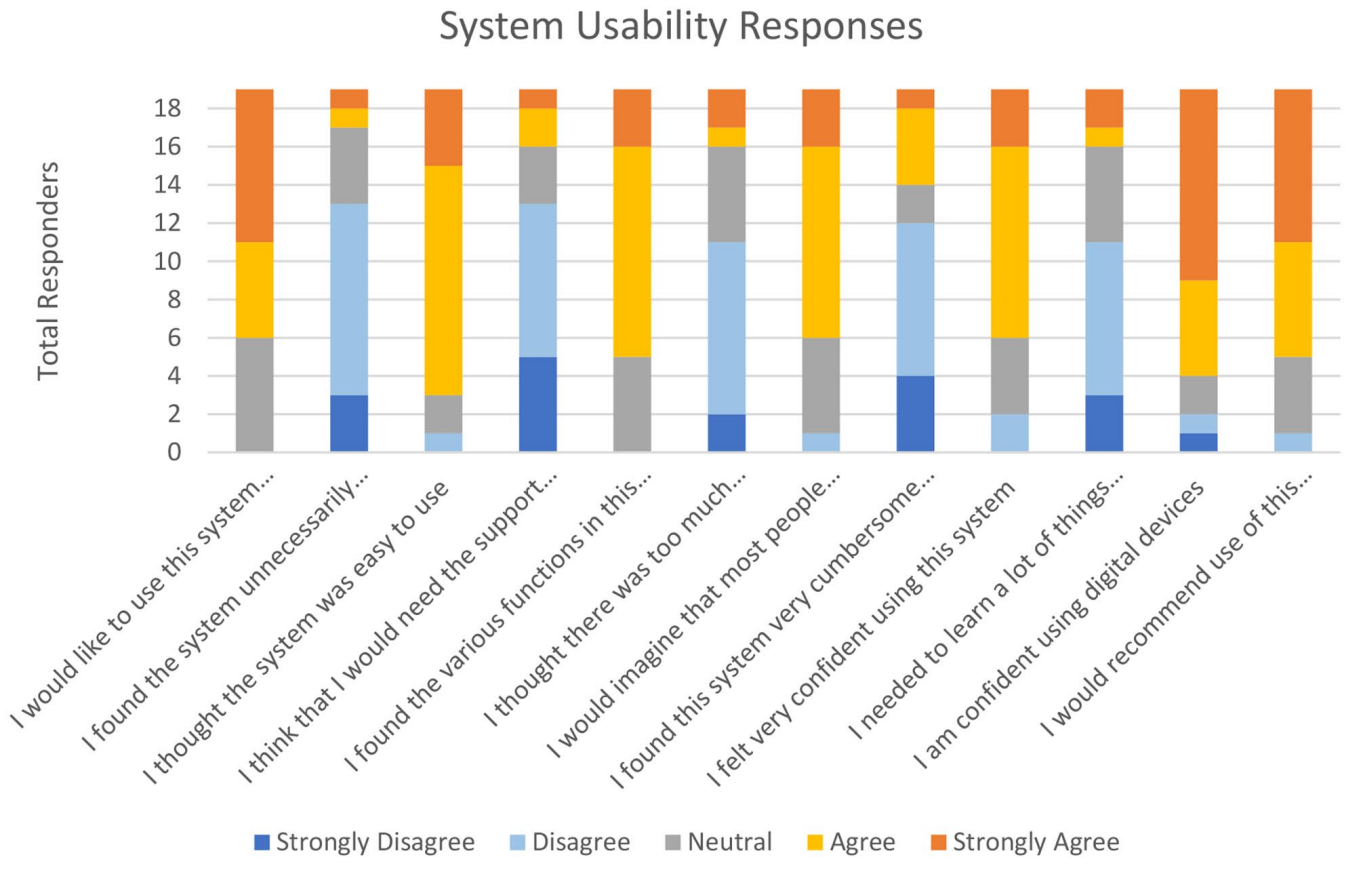
SUS results

A System Usability Score (SUS) questionnaire was issued to all staff who acted as scribe (Appendix 2) [15]. The demographics of those who replied are in Table 3. The results are summarised in Fig. 2.

**Table 3:** SUS responders - demographics

		Responders [*n* = 19]
Occupation		
	Major Trauma Coordinator	4 (21%)
	Nurse	4 (21%)
	Consultant	4 (21%)
	Junior Doctor	7 (37%)
Age		
	20–30 years	2 (10.5%)
	31–40 years	6 (31.5%)
	41–50 years	7 (37%)
	51–60 years	4 (21%)

The mean SUS in this study was 68.4. A mean score of 68 has been internationally validated as the standard of acceptable usability [10].

Overall, 73.7% of the 19 users who completed the questionnaire felt that they strongly agreed or agreed that they would like to use the system frequently. None disagreed or strongly disagreed. Only 10.5% felt that the TraumaApp was unnecessarily complex with 68.4% feeling that most people would learn to use the system very quickly.

Responses from Major Trauma Coordinators were reviewed as a subgroup, with 100% saying that they strongly agree that they would like to use this system frequently. 75% agreed that the various functions in the system were well integrated and100% agreed or strongly agreed that people would learn to use the system very quickly. 100% agreed or strongly agreed that they felt very confident using this system and that they would recommend use of this system in other trauma centres/units.

## Discussion

Accurate communication of a complex patient is critical to ensure good patient management, and enhanced team situational awareness through effective and clear documentation is vital to reduce morbidity and mortality [18, 19]. This is particularly difficult in the context of a busy, time-pressured major trauma call with continuous collection of new information [20]. Concise and contemporaneous documentation allows accurate communication between specialist teams, which has been shown to improve patient care and reduce errors [7].

Previous study of the Standby and Handover sections of this app in a simulated setting has shown equivalence in the completeness of documentation in this app versus paper proforma [14]. In this study comparing paper vs. TraumaApp during live clinical cases, no statistically significant difference between the two study groups was found. Accuracy across the different sections of the app was similar, with ‘Preparation’ carrying the lowest percentage of completeness and ‘Handover’ the highest in both groups.

Although there was a full range of ISS scores there were only two cases in which the ISS was above 15, the recognised value to define major trauma [21]. It would be beneficial to include more cases with a larger variety of ISS scores for future assessment of the TraumaApp, but this is difficult to ensure with the unpredictability of trauma cases. There were no Tier 3 trauma calls included in the study, which may be due to reduced feasibility of organising two scribes in these higher risk clinical scenarios. However, the cases encompassed a wide age range and had a ratio of 7:3 male to female which is representative of the cohort of European injury incidence in the World Health Statistics 2022 [22]. It is not representative of the overall ratio of male to female cases in the West of Scotland in 2021 [17].

There was missing data regarding the identities of participating scribes on retrospective examination of the TraumaApp documentation, but this has since been included in the routine data collation for the app, so would be more readily included in future study.

Other studies have shown electronic notes to be superior with Angotti et al. stating that completion of electronic documentation compared with paper by section was statistically significantly higher for admitting data (119.7%), pre-hospital (116.2%), primary survey (109.6%) and secondary survey (125.5%).^9^ Grundgeiger et al. showed electronic note-taking improved precision of intervention time documentation by 78%, compared to paper documentation [16]. Coffey et al. stated electronic documentation more frequently captured five data elements: time of team activation (100% vs. 85%), primary assessment (94% vs. 88%), arrival time of attending physician (98% vs. 93.5%) and disposition (100% vs. 89.5%) [23]. This has not been demonstrated in our study, but with a comparatively smaller cohort of cases.

In general, the responses from the system usability score questionnaire were encouraging: 68% of participants strongly agree or agree that they would like to use this system frequently, 84% of participants agree the system is easy to use, and 74% of participants strongly agree or agree they would recommend use of this system in other trauma centres/units. SUS was used in previous assessment of this TraumaApp [14] with an overall score of 75, which has now reduced to 68.4. This is still above the internationally validated standard of acceptable usability of 68. This reduction in score is to be expected when moving from simulated scenarios to clinical implementation, potentially reflecting the learning curve experienced by clinicians using the app. When assessing the subgroup of major trauma coordinators, who are generally more familiar with TraumaApp use, the overall satisfaction with TraumaApp usability appeared higher, with 100% recommending the app for use in other centres. This may be due to familiarity which has been demonstrated in study of other digital systems to improve operator efficiency and therefore possibly user satisfaction [24].

Feedback from staff was encouraging but shows areas for future development to improve user interface and reflecting further future training requirements. All MTC Coordinators had used the Trauma App more than 30 times each but their responses to “I feel confident using digital devices” were split with 50% strongly agreeing and 50% neutral.

Other studies have expanded the use of electronic documentation apps to pre-hospital settings or to include guidelines, such as ATLS and ETC guidelines in trauma management [9, 16].

Poor quality documentation is associated with higher patient mortality [8]. Digital devices can increase the completeness of clinical documentation. It is anticipated that the TraumaApp will be used in multiple trauma centres, leading to improved inter-hospital communication and patient care.

### Limitations

There was a limited number of patients with both paper and electronic notes available. This was attributed to a difficulty in finding two available staff members to act as scribe. Initially there was a higher proportion of paper notes but, as familiarity grew with the Trauma App, there were fewer paper notes available for comparison.

A further limitation was the study design. This was a retrospective study of patient notes. There was no randomisation or blinding of scribe participants, and as scribe participants were able to choose whether to use paper or TraumaApp this gives risk of selection bias. Included cases were not closely representative of the cohort of trauma cases seen throughout the Scottish Trauma Network in 2021.

The study did not assess the impact of familiarity with the Trauma App against improved accuracy of data recording over time, although accuracy was anticipated to improve with familiarisation.

## Conclusion

The study demonstrates equivalence in the completeness of recording patient notes on paper and with the TraumaApp. Despite this, there is growing use of the TraumaApp and usability scores are encouraging.

This study would benefit from a larger number of cases and a study of the effects on accuracy of note taking with familiarisation with the TraumaApp over time. A further study could also assess accuracy of digital documentation using video recordings of the trauma case.

It has been demonstrated that it is feasible to introduce digital data recording in major trauma assessment and management without detriment. This allows us to utilise the information sharing benefits without increasing the time required for documentation.

### Electronic supplementary material

Below is the link to the electronic supplementary material.

Supplementary Material 1

Supplementary Material 2

## Data Availability

The datasets used and/or analysed during the current study are available from the corresponding author on reasonable request.

## Abbreviations

STN: Scottish Trauma Network
MTC: Major Trauma Centre
QEUH: Queen Elizabeth University Hospital
TARN: Trauma Audit and Research Network
STAG: Scottish Trauma Audit Group
ED: Emergency Department
EHR: Electronic Health Record
SUS: System Usability Score
ISS: Injury Severity Scores
