# Supplementary material for: Implementation of major trauma app: usability and data completeness
Source: BMC Emerg Med. 2024 Jul 29;24:136. doi: 10.1186/s12873-024-01022-w (PMC11288075; doi:10.1186/s12873-024-01022-w)
Supplement: Supplementary file 1 — Supplementary Material 1 [file 12873_2024_1022_MOESM1_ESM.docx]

**Completeness Protocol TraumaApp vs paper proforma**

Errors include mistakes in details, but also the omission of information in data points listed – eg. type of trauma call activated not correct/ not documented

SECTION 1: Preparations

|  | **TraumaApp** | | **Paper** | |
| --- | --- | --- | --- | --- |
|  | **Entered/ Not entered/ NA** | **Errors** | **Entered/ Not entered/ NA** | **Errors** |
| Trauma Call Activation |  |  |  |  |
| Prepare Airway |  |  |  |  |
| ?Code red/ MHP |  |  |  |  |
| Prepare USS |  |  |  |  |
| Consider analgesia |  |  |  |  |
| Prepare Trauma Mattress |  |  |  |  |
| Appropriate PPE |  |  |  |  |
| Allocate Team |  |  |  |  |
| **Total complete / 8** |  |  |  |  |

SECTION 2: Handovers

|  | **TraumaApp** | | **Paper** | |
| --- | --- | --- | --- | --- |
|  | **Entered/ Not entered/ NA** | **Errors** | **Entered/ Not entered/ NA** | **Errors** |
| Age |  |  |  |  |
| Sex |  |  |  |  |
| Time of Injury |  |  |  |  |
| Time of Arrival |  |  |  |  |
| Mechanism |  |  |  |  |
| Injuries |  |  |  |  |
| Observations |  |  |  |  |
| Interventions |  |  |  |  |
| **Total complete / 8** |  |  |  |  |

SECTION 3: History (AMPLE)

|  | **TraumaApp** | | **Paper** | |
| --- | --- | --- | --- | --- |
|  | **Entered/ Not entered/ NA** | **Errors** | **Entered/ Not entered/ NA** | **Errors** |
| Allergies |  |  |  |  |
| Medication |  |  |  |  |
| PMH |  |  |  |  |
| Last ate/ drank |  |  |  |  |
| **Total complete /4** |  |  |  |  |

Note: Unable to assess “events leading” as not on paper proforma

SECTION 4: Primary Survey

|  | **TraumaApp** | | **Paper** | |
| --- | --- | --- | --- | --- |
|  | **Entered/ Not entered/ NA** | **Errors** | **Entered/ Not entered/ NA** | **Errors** |
| <C> |  |  |  |  |
| C-spine immobilisation |  |  |  |  |
| A |  |  |  |  |
| Status ie clear/ compromised |  |  |  |  |
| Interventions |  |  |  |  |
| B |  |  |  |  |
| Breathing exam |  |  |  |  |
| Observations |  |  |  |  |
| Interventions |  |  |  |  |
| C |  |  |  |  |
| Circulation Exam |  |  |  |  |
| Observations |  |  |  |  |
| Interventions |  |  |  |  |
| USS |  |  |  |  |
| POC - VBG |  |  |  |  |
| D |  |  |  |  |
| GCS |  |  |  |  |
| Neuro status |  |  |  |  |
| Pupillary response |  |  |  |  |
| E |  |  |  |  |
| Blood Glucose |  |  |  |  |
| Temperature |  |  |  |  |
| Interventions |  |  |  |  |
