# Supplementary material for: Implementation of major trauma app: usability and data completeness
Source: BMC Emerg Med. 2024 Jul 29;24:136. doi: 10.1186/s12873-024-01022-w (PMC11288075; doi:10.1186/s12873-024-01022-w)
Supplement: Supplementary file 2 — Supplementary Material 2 [file 12873_2024_1022_MOESM2_ESM.docx]

System Usability Scale

© Digital Equipment Corporation 1986

|  | Strongly Disagree | Disagree | Neutral | Agree | Strongly Agree |
| --- | --- | --- | --- | --- | --- |
| I would like to use this system frequently |  |  |  |  |  |
| I found the system unnecessarily complex |  |  |  |  |  |
| I thought the system was easy to use |  |  |  |  |  |
| I think that I would need the support of a technical person to use this system |  |  |  |  |  |
| I found the various functions in this system were well integrated |  |  |  |  |  |
| I thought there was too much inconsistency in this system |  |  |  |  |  |
| I would imagine that most people would learn to use this system very quickly |  |  |  |  |  |
| I found this system very cumbersome to use |  |  |  |  |  |
| I felt very confident using this system |  |  |  |  |  |
| I needed to learn a lot of things before I could get going with this system |  |  |  |  |  |
| I am confident using digital devices |  |  |  |  |  |
| I would recommend use of this system in other trauma centres/units |  |  |  |  |  |
